# Supplementary material for: A-to-I miR-378a-3p editing can prevent melanoma progression via regulation of PARVA expression
Source: Nat Commun. 2018 Jan 31;9:461. doi: 10.1038/s41467-018-02851-7 (PMC5792646; doi:10.1038/s41467-018-02851-7)

**SUPPLEMENTARY FIGURES**

**
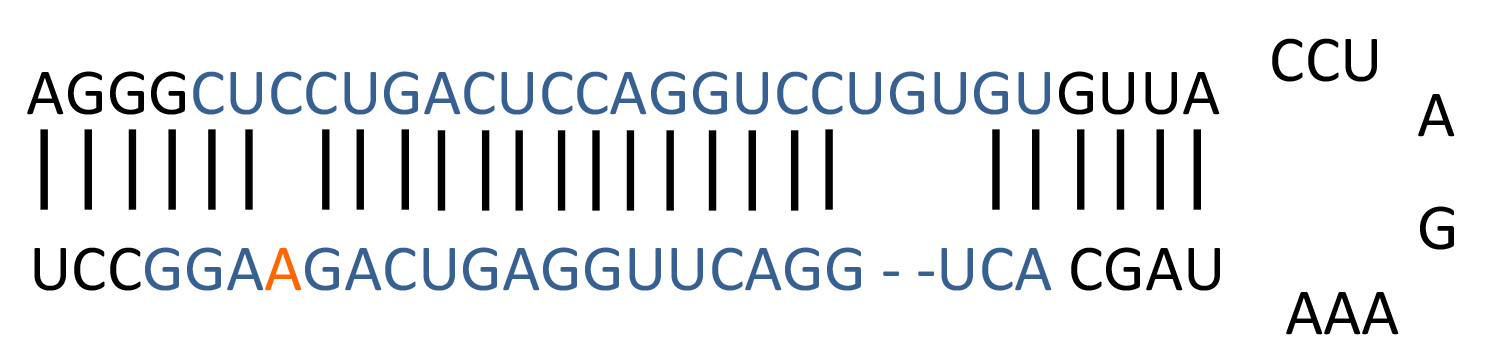
**

**Supplementary Figure 1: miR-378a-3p editing in melanoma.** Blue sequence represents mature miR-378a-3p and the editing site, at position 18 (non-canonical seed region), highlighted in orange. This data was obtained by deep RNA sequencing as previously describe in Shoshan, E. *et al*.^8^

**
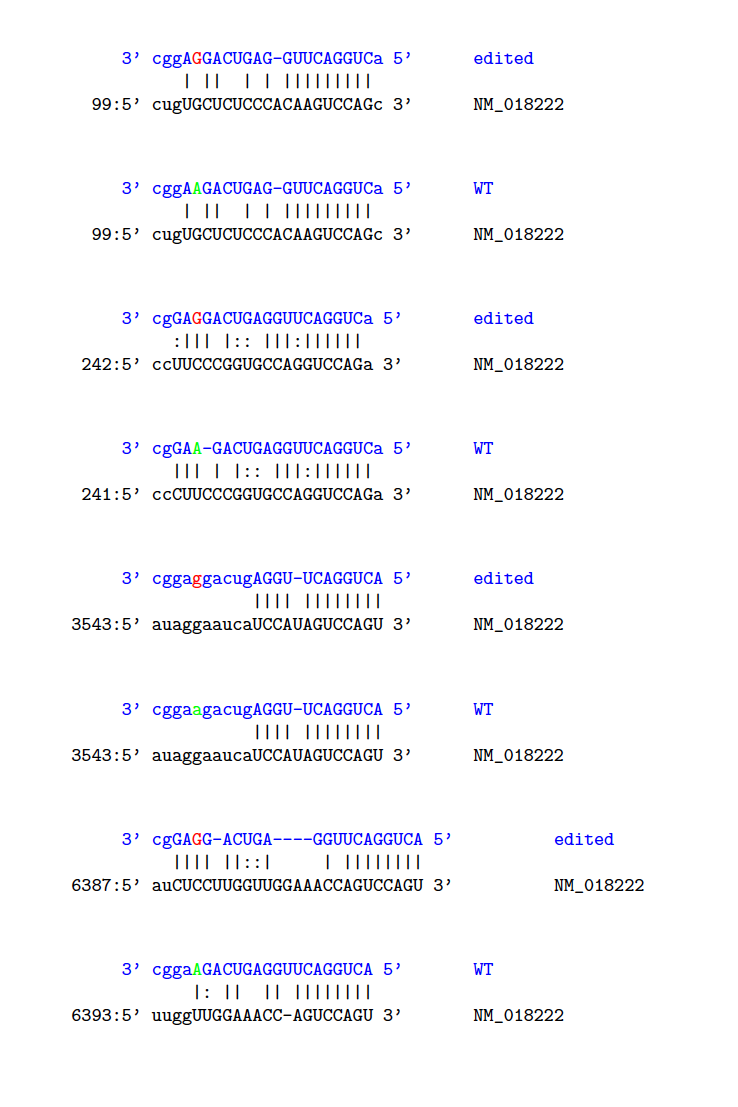
**

**Supplementary Figure 2: Binding Prediction of Edited or Wild-Type miR-378a-3p to *PARVA*.** Utilization of perl am latex on miRanda, PITA, RNA hybrid analyses for

## miR-378a-3p WT and edited versus *PARVA* (NM_018222) to demonstrate the predicted binding site of miR-378a-3p on the 3’UTR of *PARVA*.

##

## **Supplementary Figure 3. Edited miR-378a-3p down-regulated *PARVA* mRNA expression.** The effect of edited miR-378a-3p on the *PARVA* mRNA levels were measured via RT-qPCR in transfected C8161 cells (left panel) and SB2 cells (right panel).

**
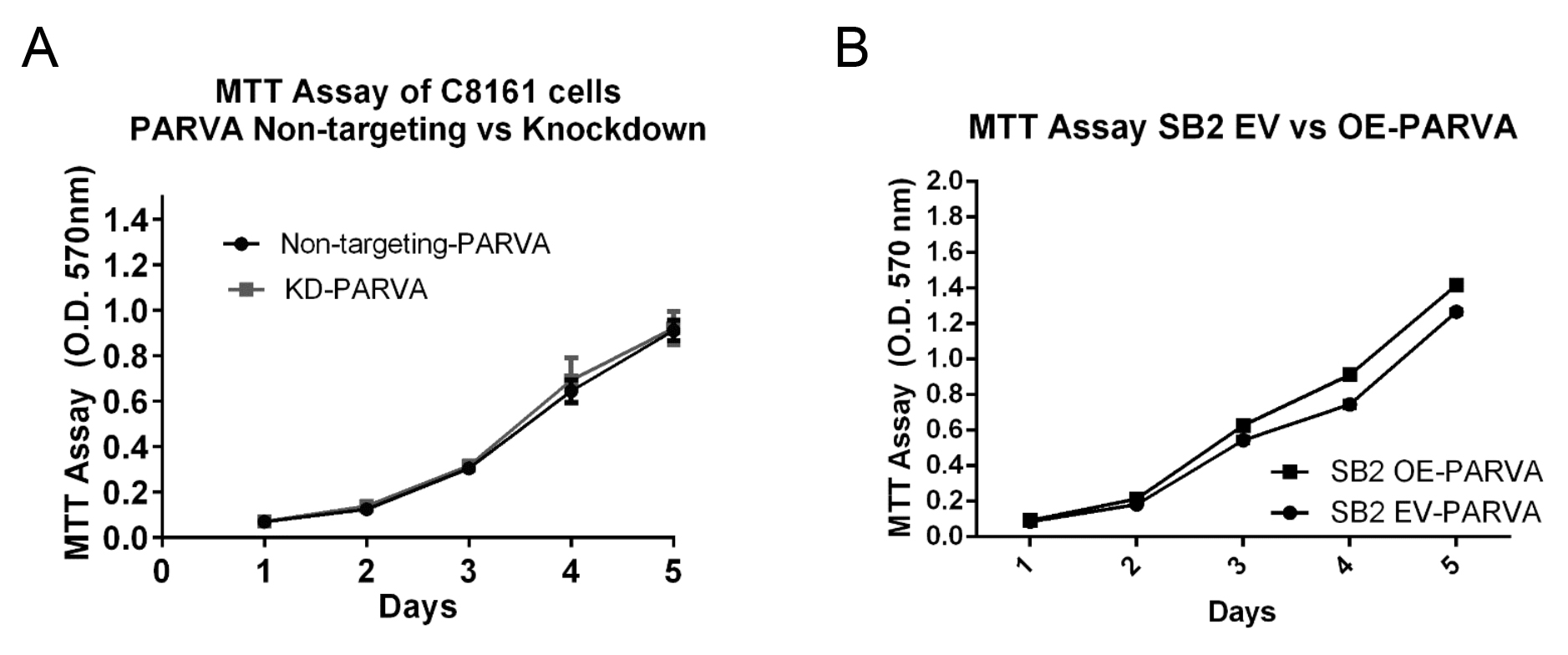
**

**Supplementary Figure 4: Effect of *PARVA* silencing or overexpression on melanoma cell viability.** MTT assays were performed to determine doubling time of

## **(A)** C8161 after *PARVA* silencing and **(B)** SB2 after *PARVA* overexpression. No significant changes in cell proliferation were observed.

##
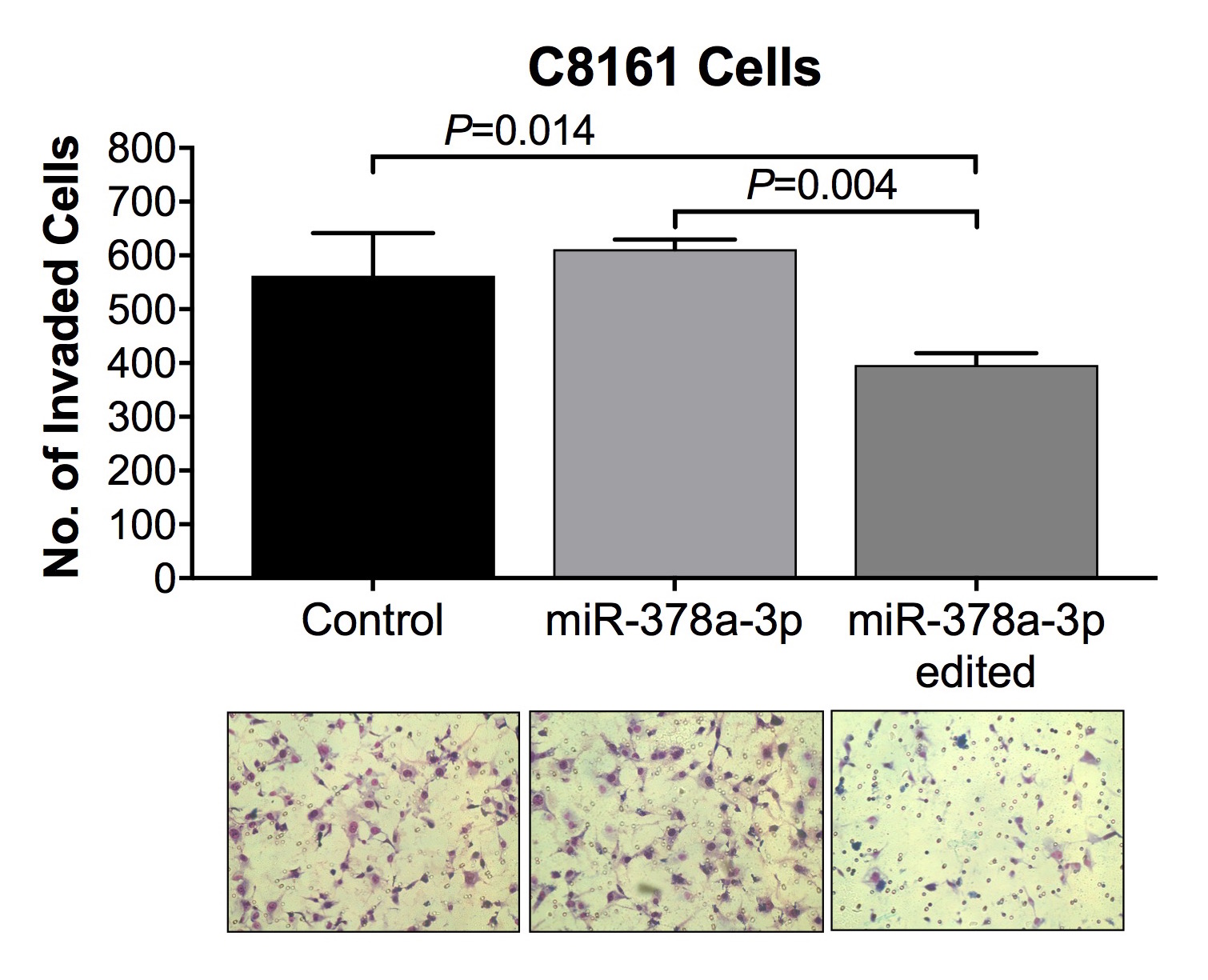


## **Supplementary Figure 5. Edited miR-378a-3p decreases cell invasion.**

## C8161 cells transfected with edited miR-378a-3p showed a significant reduction in the number of invaded cells (*P*=0.014) compared to scramble control. The data are shown as the means ± S.D., n=3.

## **Supplementary Figure 6.**
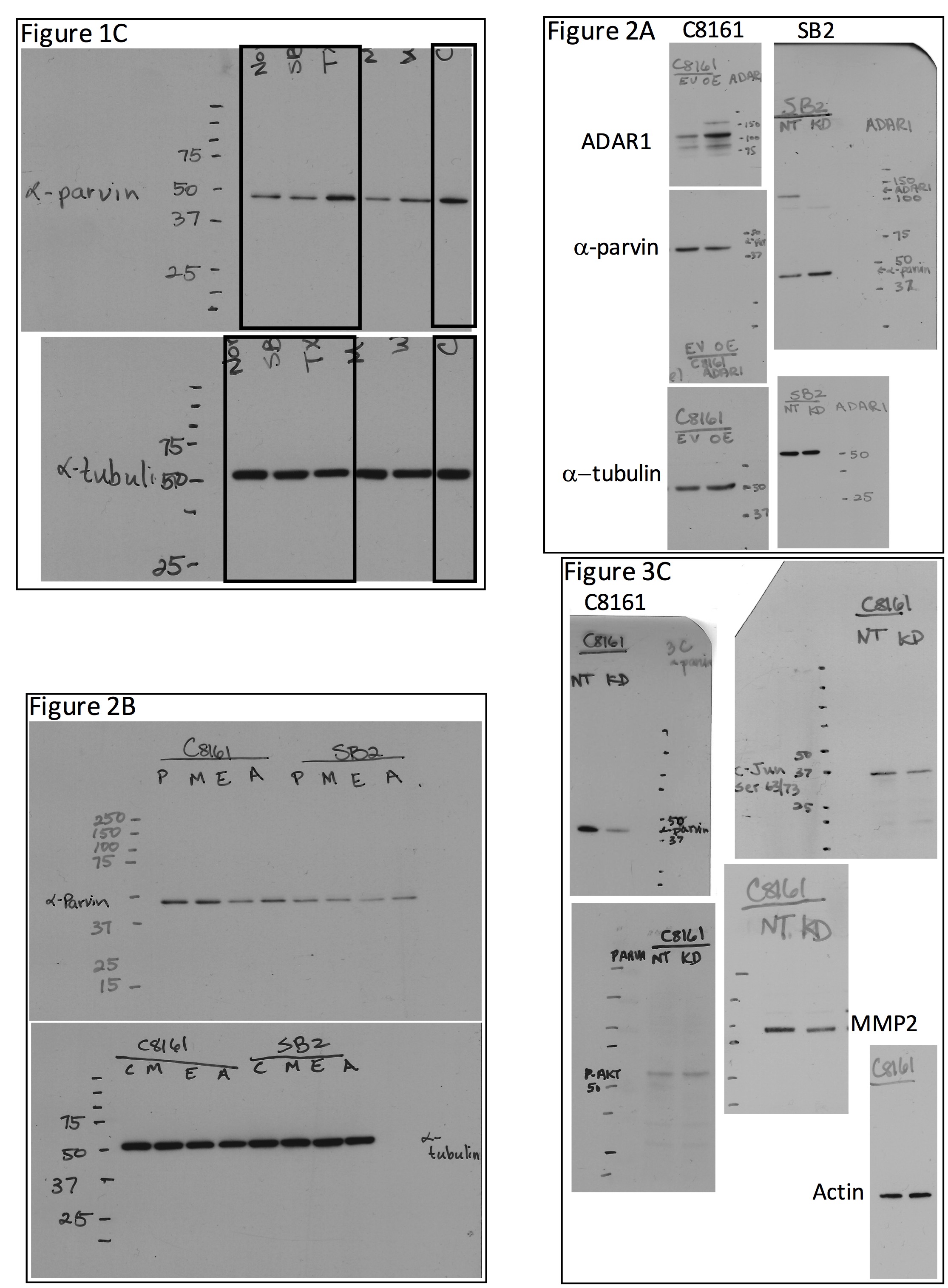
**Full Blots**

##

## **SUPPLEMENTARY TABLES**

## **Supplementary Table 1. Univariate analysis (overall survival)**

##
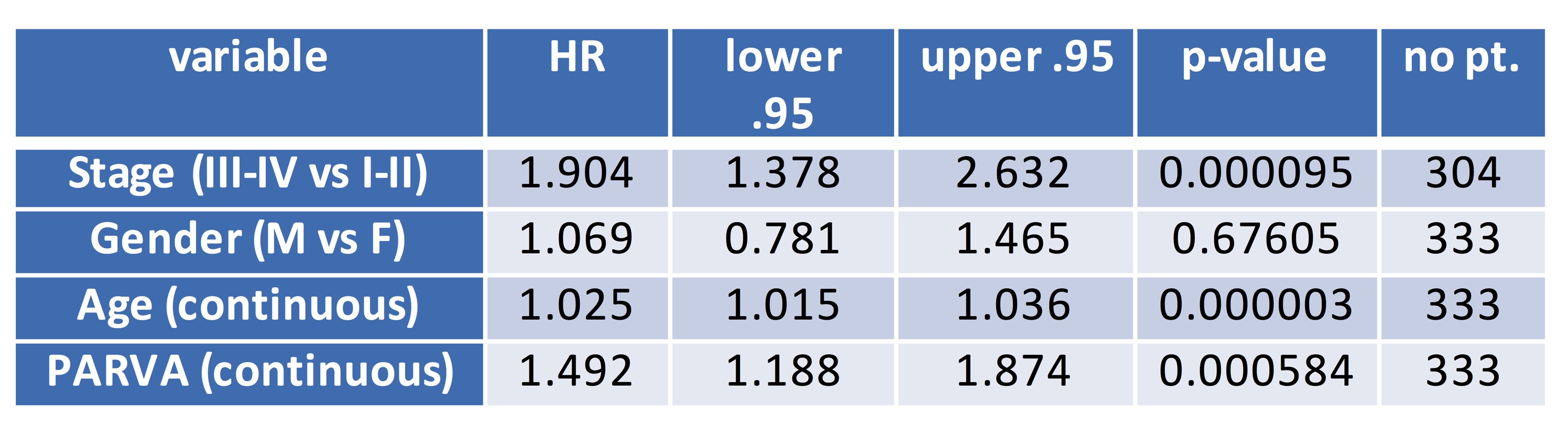


## **Supplementary Table 2. Multivariate analysis (overall survival): Stage+Age+mRNA**

##
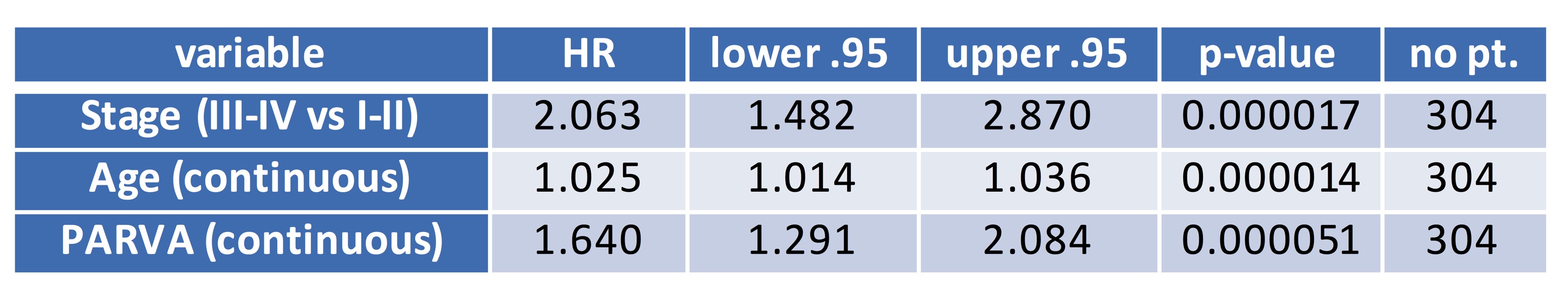

Supplement: Supplementary file 1 — Supplementary Information [file 41467_2018_2851_MOESM1_ESM.docx]
