# Supplementary material for: A-to-I miR-378a-3p editing can prevent melanoma progression via regulation of PARVA expression
Source: Nat Commun. 2018 Jan 31;9:461. doi: 10.1038/s41467-018-02851-7 (PMC5792646; doi:10.1038/s41467-018-02851-7)
Supplement: Supplementary file 3 — Description of Additional Supplementary Files [file 41467_2018_2851_MOESM3_ESM.pdf]

Description of Additional Supplementary Files

File Name: Supplementary Data 1
Description: Differential gene expression (Log-ratio 0.5, $p<0.01$) using Nexus expression 3.
