# Supplementary material for: A-to-I miR-378a-3p editing can prevent melanoma progression via regulation of PARVA expression
Source: Nat Commun. 2018 Jan 31;9:461. doi: 10.1038/s41467-018-02851-7 (PMC5792646; doi:10.1038/s41467-018-02851-7)
Supplement: Supplementary file 4 — Supplementary Data 1 [file 41467_2018_2851_MOESM4_ESM.pdf]

Supplementary Data 1. Differential gene expression (Log-ratio 0.5, p<0.01) using Nexus expression 3

Comparison 1 - Groups - <Edited> vs. Avg of <Mimic> (175:131)
#Log-Ratio Threshold - 0.5
#P-Value Threshold - 0.01
#Multiple Test Correction - None

Probe	Gene Symbol	regulation	log-ratio	p-value
1553055_a_at	SLFN5	DOWN	-0.7591	0.0005
1559883_s_at	SAMHD1	DOWN	-1.0074	0.0002
1568592_at	TRIM69	DOWN	-0.5875	0.0028
200887_s_at	STAT1	DOWN	-0.7222	0.0011
201469_s_at	SHC1	DOWN	-0.5685	0.0024
201502_s_at	NFKBIA	DOWN	-0.518	0.0002
201601_x_at	IFITM1	DOWN	-1.21	0.0015
201649_at	UBE2L6	DOWN	-0.7147	0.0019
201708_s_at	NIPSNAP1	DOWN	-0.5133	0.0037
202145_at	LY6E	DOWN	-0.5289	0.0008
202217_at	C21orf33	DOWN	-1.1039	0.0001
202411_at	IFI27	DOWN	-2.2919	0.0001
202430_s_at	PLSCR1	DOWN	-0.74	0.0021
202446_s_at	PLSCR1	DOWN	-0.6745	0.0005
202638_s_at	ICAM1	DOWN	-0.588	0.0035
202663_at	WIPF1	DOWN	-0.6412	0.0021
202869_at	OAS1	DOWN	-1.9323	0.0009
203147_s_at	TRIM14	DOWN	-0.5115	0.0005
203153_at	IFIT1	DOWN	-1.5449	0.0012
203882_at	ISGF3G	DOWN	-0.8991	0.0003
203974_at	HDHD1A	DOWN	-0.8698	0.0069
204211_x_at	EIF2AK2	DOWN	-0.5922	0.0074
204415_at	IFI6	DOWN	-2.0997	0.0001
204439_at	IFI44L	DOWN	-1.6436	0.0013
204470_at	CXCL1	DOWN	-0.5737	0.0009
204502_at	SAMHD1	DOWN	-1.0912	0.0024
204698_at	ISG20	DOWN	-0.7921	0.0031
204747_at	IFIT3	DOWN	-1.2083	0.0006
204909_at	DDX6	DOWN	-0.5427	0.0005
204972_at	OAS2	DOWN	-1.6041	0.007
205067_at	IL1B	DOWN	-0.5978	0.0071
205100_at	GFPT2	DOWN	-1.2487	0.0002
205483_s_at	ISG15	DOWN	-1.389	0.0002
205552_s_at	OAS1	DOWN	-2.5763	0
205660_at	OASL	DOWN	-1.5254	0.0003
206133_at	XAF1	DOWN	-1.1211	0.0017
208012_x_at	SP110	DOWN	-0.9544	0.0014
208436_s_at	IRF7	DOWN	-1.2089	0.0028
208965_s_at	IFI16	DOWN	-0.5412	0.0006
208996_s_at	POLR2C	DOWN	-0.5136	0.0001
209417_s_at	IFI35	DOWN	-0.9711	0.0026
209518_at	SMARCD1	DOWN	-0.7969	0.0044
209761_s_at	SP110	DOWN	-1.2139	0.0017
209762_x_at	SP110	DOWN	-1.2253	0.0006
209774_x_at	CXCL2	DOWN	-0.5867	0.0003
209852_x_at	PSME3	DOWN	-0.7006	0
209906_at	C3AR1	DOWN	-0.5019	0.0003
209969_s_at	STAT1	DOWN	-1.294	0.0012
210667_s_at	C21orf33	DOWN	-1.3287	0.0003
210797_s_at	OASL	DOWN	-1.2505	0.0017
211506_s_at	IL8	DOWN	-0.6532	0.0008
212203_x_at	IFITM3	DOWN	-0.5534	0.0003
213015_at		DOWN	-0.6788	0.0038
213043_s_at	MED24	DOWN	-0.8127	0.0007
213797_at	RSAD2	DOWN	-1.7779	0.0033
214022_s_at	IFITM1	DOWN	-1.7257	0.0003
214290_s_at	HIST2H2AA3	DOWN	-0.6336	0.005
214374_s_at	PPFIBP1	DOWN	-0.55	0.009
214453_s_at	IFI44	DOWN	-0.7456	0.0006
215247_at	LOC440895	DOWN	-0.5432	0.0018
215411_s_at	TRAF3IP2	DOWN	-1.1286	0.0005
216565_x_at	LOC391020	DOWN	-0.6618	0.0004
217502_at	IFIT2	DOWN	-0.7109	0.0024
217839_at	TFG	DOWN	-0.9163	0
**217890_s_at**	**PARVA**	**DOWN**	**-1.3902**	**0.0001**
218145_at	TRIB3	DOWN	-0.6652	0.0063
218400_at	OAS3	DOWN	-1.2565	0.0027

Probe	Gene Symbol	regulation	log-ratio	p-value
1553603_s_at	ARL6IP2	UP	0.6592	0.0012
1554020_at	BICD1	UP	0.631	0.0015
1559006_at		UP	0.6911	0.0059
200998_s_at	CKAP4	UP	0.5401	0.0012
201677_at	C3orf37	UP	0.6334	0.0088
201710_at	MYBL2	UP	0.567	0.0029
201896_s_at	PSRC1	UP	0.5486	0.0038
202084_s_at	SEC14L1	UP	0.5351	0.0066
202094_at	BIRC5	UP	0.5767	0.0012
202743_at	PIK3R3	UP	0.5435	0.0078
203284_s_at	HS2ST1	UP	0.517	0.0088
203537_at	PRPSAP2	UP	0.5147	0.0083
204727_at	WDHD1	UP	0.6168	0.0039
204728_s_at	WDHD1	UP	0.6438	0.0019
205474_at	CRLF3	UP	1.0463	0.003
211814_s_at	CCNE2	UP	0.5317	0.0008
212836_at	POLD3	UP	0.5132	0.0011
213032_at	NFIB	UP	0.6456	0.0075
214820_at	BRWD1	UP	0.752	0.0089
218031_s_at	FOXN3	UP	0.5755	0.0046
219226_at	CRKRS	UP	0.7015	0.0004
219531_at	CEP72	UP	0.6715	0.0082
221488_s_at	CUTA	UP	0.5648	0.0001
222734_at	WARS2	UP	0.5016	0.0083
223214_s_at	ZHX1	UP	0.5579	0.0014
223595_at	TMEM133	UP	0.5726	0.005
224521_s_at	CCDC77	UP	0.5078	0.01
225205_at	KIF3B	UP	0.8722	0.0014
226265_at	QSER1	UP	0.6188	0.0013
226413_at	LOC400027	UP	0.549	0.0085
226829_at	AFAP1L2	UP	0.6103	0.0025
227395_at		UP	0.698	0.0019
228416_at	ACVR2A	UP	0.5069	0.0055
228920_at	ZNF260	UP	0.5804	0.0039
229114_at		UP	0.5482	0.0087
229134_at	VANGL1	UP	0.5197	0.0047
229492_at	VANGL1	UP	0.6173	0.0084
229544_at		UP	0.5081	0.0055
229674_at	SERTAD4	UP	0.5092	0.0036
230097_at	GART	UP	0.5421	0.0039
230201_at		UP	0.5969	0.0047
231975_s_at	MIER3	UP	0.54	0.0087
232291_at	MIRH1	UP	0.6624	0.0062
235470_at		UP	0.6173	0.0049
236219_at	TMEM20	UP	0.9916	0.0089
237400_at	ATP5S	UP	0.5045	0.0016
239944_at	NAIP	UP	0.5664	0.0034
242245_at		UP	0.5056	0.0048
243852_at	LUC7L2	UP	0.5268	0.0036
244563_at	QSER1	UP	0.9039	0

218699_at	RAB7L1	DOWN	-0.6547	0.005
218915_at	NF2	DOWN	-1.2066	0
218943_s_at	DDX58	DOWN	-1.1235	0.0005
218984_at	PUS7	DOWN	-0.5209	0.0016
219209_at	IFIH1	DOWN	-1.1848	0.0066
219352_at	HERC6	DOWN	-1.446	0.0016
219863_at	HERC5	DOWN	-0.6024	0.0009
221735_at	WDR48	DOWN	-0.9761	0.0011
221766_s_at	FAM46A	DOWN	-0.637	0.0003
**222455_s_at**	**PARVA**	**DOWN**	**-0.8465**	**0.0009**
222793_at	DDX58	DOWN	-0.9199	0.0029
222907_x_at	TMEM50B	DOWN	-0.5044	0.0012
222995_s_at	RHBDD2	DOWN	-0.7959	0.0001
223218_s_at	NFKBIZ	DOWN	-0.6616	0.0066
223220_s_at	PARP9	DOWN	-1.334	0.0001
223266_at	ALS2CR2	DOWN	-0.559	0
223849_s_at	MOV10	DOWN	-0.7087	0.0033
223888_s_at	LARS	DOWN	-0.5981	0.002
224354_at		DOWN	-0.9174	0.0061
224559_at	MALAT1	DOWN	-1.936	0.0091
224851_at	CDK6	DOWN	-0.7364	0.0043
225188_at	RAPH1	DOWN	-0.6108	0.0004
225189_s_at	RAPH1	DOWN	-0.5718	0.0016
225415_at	DTX3L	DOWN	-0.5861	0
225616_at	SPRYD4	DOWN	-0.6495	0.0007
225636_at	STAT2	DOWN	-0.5688	0.0022
225804_at	CYB5D2	DOWN	-0.5698	0.0011
226603_at	SAMD9L	DOWN	-1.0852	0.0018
226702_at	LOC129607	DOWN	-1.5109	0.001
226734_at	EIF4E2	DOWN	-0.6104	0.0014
226757_at	IFIT2	DOWN	-0.5948	0.0048
227309_at	YOD1	DOWN	-1.004	0.0001
227807_at	PARP9	DOWN	-0.6825	0.0006
228152_s_at	FLJ31033	DOWN	-0.6423	0.0076
228230_at	PRIC285	DOWN	-0.681	0.0066
228607_at	OAS2	DOWN	-1.5804	0.0038
228617_at	XAF1	DOWN	-0.9418	0.0058
229450_at	IFIT3	DOWN	-0.9982	0.0003
230314_at		DOWN	-0.9081	0.0001
231835_at	C1orf93	DOWN	-0.5528	0.0005
233917_s_at	MOV10	DOWN	-0.606	0.0041
234987_at		DOWN	-0.975	0.0016
235529_x_at		DOWN	-0.9286	0.0004
235643_at	SAMD9L	DOWN	-0.6179	0.0095
235964_x_at		DOWN	-0.8344	0.0001
239038_at	C1orf52	DOWN	-0.7141	0.0006
242625_at	RSAD2	DOWN	-2.1558	0.001
242961_x_at	DDX58	DOWN	-0.8805	0.0032
243271_at		DOWN	-0.606	0.0001
244881_at	LMLN	DOWN	-0.6793	0.0055
39402_at	IL1B	DOWN	-0.5434	0.0096
52164_at	C11orf24	DOWN	-0.5662	0.0014
53720_at	FLJ11286	DOWN	-0.5103	0.0008
56919_at	WDR48	DOWN	-0.7701	0.0002
AFFX-HUMISGF3A/M97935_3_at	STAT1	DOWN	-0.7796	0.0024
AFFX-HUMISGF3A/M97935_5_at	STAT1	DOWN	-1.2691	0.0007
AFFX-HUMISGF3A/M97935_MA_at	STAT1	DOWN	-1.1665	0.0001
AFFX-HUMISGF3A/M97935_MB_at	STAT1	DOWN	-0.8872	0.002
